# The Increasing Level of DKK-1 as a New Bone Formation Factor in Patients with Early Spondyloarthritis

**DOI:** 10.1155/2023/5543234

**Published:** 2023-05-23

**Authors:** Yuliasih Yuliasih, Aghnia Permatasari, Lita Diah Rahmawati, Mohammad Imam Wahyudi, Nabilatun Nisa'

**Affiliations:** ^1^Rheumatology Division, Internal Medicine Department, Airlangga University, Surabaya 60132, Indonesia; ^2^Immunology, Postgraduate School, Universitas Airlangga, Surabaya 60286, Indonesia; ^3^Internal Medicine Department, Faculty of Medicine, Airlangga University, Surabaya 60132, Indonesia; ^4^Department of Biology, Faculty of Science and Technology, Airlangga University, Surabaya 60115, Indonesia

## Abstract

The role of dickkopf-related protein 1 (DKK-1) in radiographic development may become a robust marker for early spondyloarthritis (SpA) diagnosis. This study aimed at determining the serum DKK-1 profile in patients with SpA and investigating its relationship with SpA progression. Supported by analyzing the BMD data which aims to affirm the potential of DKK-1 as a biomarker for early diagnosis of SpA, this research may become the early study to produce a robust tool to diminish the fatal impacts in SpA. This cross-sectional study included patients with SpA using ASAS 2010 criteria from Dr. Soetomo General Hospital, Indonesia. Collected data included patients' general characteristics, disease duration, disease activity using ASDAS-CRP and ASDAS-ESR, serum DKK-1 levels, and BMD. The patients were classified as early SpA if the disease duration was ≤5 years and established SpA if the disease duration was >5 years, while the low BMD was indicated by *Z* score ≤ −2.00. The correlation was tested using the Spearman or Pearson test. The differences in patients' characteristics among early and established SpA and also between low and normal BMD were tested using the unpaired *T*-test or the Mann–Whitney test. The serum DKK-1 levels in early SpA (7365 ± 2067 pg/dL) were significantly higher than those in established SpA (5360 ± 1054 pg/dL). Serum DKK-1 levels were also associated with disease duration (*r* = −0.370, *p* = 0.040) and BMD at the total hip (*r* = 0.467, *p* = 0.028). The differences in all patients' clinical parameters were not found between patients with low BMD at any site and patients with normal BMD unless in the BMI (*p* = 0.019). Our findings found DKK-1 as a potential diagnostic marker for early SpA. Early diagnosis may lead to rapid treatment to delay disease progression and prevent future impairment.

## 1. Introduction

Spondyloarthritis (SpA) is a group of chronic inflammatory diseases that are phenotypically distinct but linked, including psoriatic arthritis, reactive arthritis, enteropathic arthritis, and ankylosing spondylitis, and are characterised by enthesitis, sacroiliitis, and axial joint involvement [1]. Most patients with SpA suffer from a combination of inflammation and structural bone damage [2]. In SpA, structural damage manifests as new bone development that results in syndesmophytes, bone bridges, and total ankylosis [3]. Ankylosis, which alters the balance of loads and forces on the skeletal system and causes muscular inflexibility and an accelerated degenerative spine disorder, is the primary effect of structural damage that affects patients with SpA and renders them disabled, permanently robbed of their function [4].

Early diagnosis allows rapid and effective therapy to be started to avoid the worse deformities, stiffness, and restrictive respiratory illness found in severe SpA [5]. But there is difficulty in diagnosing SpA at an early stage. For example, the back pain and discomfort at tendon insertion sites are quite prevalent and considered minor gripe in the general population. In addition, the radiographic alterations take a longer period to manifest [6]. Specific markers for detecting SpA early will enormously assist the SpA treatment in preventing patients from further physical disabilities.

The serum dickkopf-related protein 1 (DKK-1) levels and radiographic development in axial SpA patients are associated [7]. A study using the animal arthritis model revealed that DKK-1 blockage causes the sacroiliac joints to fuse [8], and high levels of functioning DKK-1 appear to be protective of the development of syndesmophytes [9]. Patients with axial SpA have reported reduced serum DKK-1 levels compared to healthy controls [10] and patients with other rheumatic inflammatory illnesses such as rheumatoid arthritis [11]. In addition to DKK-1, the formation of new syndesmophytes in young axial SpA patients was also correlated with low bone mineral density (BMD) via a systemic inflammatory process that altered systemic bone remodelling [12, 13]. Although studies of the correlation between DKK-1 and BMD in SpA have already been reported, there are scant studies comparing these two factors simultaneously, particularly in SpA patients from Indonesia. The major genetic involvement in autoimmune disease, particularly SpA, enables regional variations in disease appearance and development [14].

The purpose of this study was to reveal the profile of serum DKK-1 in patients with SpA at Dr. Soetomo General Hospital, Surabaya, Indonesia, and to analyze its relationship with SpA progression. Supported by analyzing the BMD data which aims to affirm the potential of DKK-1 as a biomarker for early diagnosis of SpA, this research may become the early study to produce a robust tool to diminish the fatal impacts in SpA.

## 2. Materials and Methods

### 2.1. Patients and Study Design

This cross-sectional study included 31 patients with early (≤5 years) and established SpA (>5 years) [15] from the Dr. Soetomo General Hospital Rheumatology Outpatient Clinic that met the Assessment of Spondyloarthritis International Society (ASAS) 2010 criteria. The sample size calculation was performed using the formula for cross-sectional studies for correlative analysis. Patients who had a history of smoking, alcohol consumption, hormonal contraceptive use, vitamin D and/or calcium supplementation, glucocorticoid therapy, chronic kidney disease, liver cirrhosis, diabetes mellitus, malignancy, infection, menopause, and other autoimmune diseases were excluded. This study has been carried out in accordance with the Code of Ethics of the World Medical Association (Declaration of Helsinki) for experiments involving humans. Informed consent was obtained prior to participation in this study, and the confidentiality of all patients' information was guaranteed. The study was approved by Dr. Soetomo hospital's Research Ethics Committee (number: 0357/KEPK/I/2022).

### 2.2. Clinical and Laboratory Assessment

Disease-related data and clinical scorings were collected from all patients. Ankylosing spondylitis disease activity score-C-reactive protein (ASDAS-CRP) supported by the ASDAS-erythrocyte sedimentation rate (ASDAS-ESR) was used to evaluate the disease activity. This score combines patient-reported pains, and the laboratory data included the following: back pain, duration of morning stiffness, peripheral swelling pain, and global status of SpA. The laboratory examination component assessed was CRP and ESR levels. The cutoff values for assessing the disease activity using ASDAS scores are as follows: <1.3, inactive disease; 1.3–2.1, low disease activity; 2.1–3.5, high disease activity; and >3.5, very high disease activity [16, 17].

### 2.3. Serum DKK-1 Level and BMD Evaluation

The DKK-1 levels were obtained from all patients' blood serum using the Quantikine® ELISA human DKK-1 reagent kit (R&D Systems, Inc., Minneapolis, USA) and laboratory tests were conducted in the Prodia Laboratory. The normal reference levels were 2513 pg/mL with normal range of 1357–5290 pg/mL [18]. The ELISA measurements were taken using the Microplate Reader Bio-Rad model 680 (Bio-Rad Laboratories Inc., CA, USA) with Microplate Manager software version 5.2.1 (Bio-Rad Laboratories Inc., CA, USA). The BMD of the lumbar spine and left hip was assessed using dual-energy X-ray absorptiometry (DXA) by Lunar Prodigy densitometer, Madison, WI, USA. The BMD assessment is conducted using anteroposterior projection at L1–L4 and is expressed as the number of grams of bone mineral per square centimeter (g/cm^2^), and *Z* scores were also calculated according to the manufacturer's protocols. The *Z* score ≤−2.0 is considered to be below the expected range and defined as low BMD according to the International Society of Clinical Densitometry [19].

### 2.4. Statistical Analysis

A descriptive statistic was performed to general patients' characteristics including sex, age, body mass index (BMI), disease duration, disease phenotype, ESR, CRP, ASDAS-ESR, ASDAS-CRP, serum DKK-1 levels, disease-modifying antirheumatic drugs (DMARD) or non-DMARD treatment, BMD score, *Z* score, and modified stoke ankylosing spondylitis spinal score (mSASSS). The differences in patients' characteristics among early and established SpA and also between normal BMD and low BMD at any site are analyzed using the unpaired *T*-test for normally distributed data or the Mann–Whitney test for non-normally distributed data. The Pearson correlation test (for normally distributed data) or the Spearman correlation test (for non-normally distributed data) was performed to analyze the correlation between the DKK-1 levels and patients' clinical parameters. For all statistical analyses that were two-tailed and conducted using GraphPad Prism 9 for Windows, *p* values less than 0.05 were considered statistically significant.

## 3. Results

Table 1 shows the general patients' characteristics in a total of 31 SpA patients at the Dr. Soetomo General Hospital, Surabaya, Indonesia.

The association between patients' characteristics and disease duration is shown in Table 2. The CRP, ESR, ASDAS-ESR, and ASDAS-CRP in patients with established SpA are significantly higher compared to patients with early SpA. In contrast, the serum DKK-1 levels in early SpA were significantly higher than those in established SpA, with a *p* value of 0.001.

**Figure 1:**
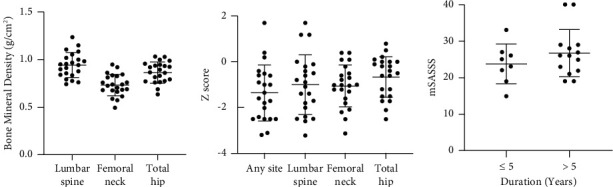
Bone mineral density and mSASSS in a total of 22 patients. (a) BMD score, (b) *Z* score, and (c) mSASSS. BMD: bone mineral density and mSASSS: modified stoke ankylosing spondylitis spinal score.

Only 22 out of the total 31 patients agreed to undergo BMD and mSASSS examination.  Figure 1 shows the score of BMD and mSASSS in the study subjects. The mean BMD at the lumbar spine, femoral neck, and total hip was 0.94 ± 0.13 g/cm^2^, 0.73 ± 0.11 g/cm^2^, and 0.87 ± 0.11 g/cm^2^, respectively. Total patients with low BMD, indicated by *Z* score ≤ −2.00, at any site were 8 patients (36.36%), at the lumbar spine were 6 patients (27.27%), at the femoral neck were 4 patients (18.18%), and at the total hip were 2 patients (9.09%). The mean mSASSS in this study was 25.64 ± 6.20.

There was no difference in all patients' parameter values between normal and low BMD, as shown in Table 3, unless the BMI was significantly lower in patients with low BMD. Although statistically insignificant, patients with low BMD were not necessarily exhibiting characteristics that suggest poor SpA progression. Patients with low BMD have a younger age average and lower BMI with lower ESR, CRP, serum DKK-1 level, and mSASSS. For disease activity, both ASDAS-ESR and ASDAS-CRP were higher in patients with normal BMD. Interestingly, patients with low BMD have shorter disease duration than patients with normal BMD.

Table 4 shows the correlation between DKK-1 levels and all patients' clinical parameters. Only duration and BMD at the total hip were correlated with DKK-1 levels. A negative *r*-value indicates a negative correlation, which means that DKK-1 levels increase significantly at an earlier SpA duration. Conversely, there was a positive correlation between DKK-1 levels and BMD at the total hip which indicates a positive *r*-value.

## 4. Discussion

In this cross-sectional study, we found 31 SpA patients with high disease activity as indicated by a mean ASDAS-CRP score of 2.43 ± 1.09. The average disease duration in this study was relatively short with an average of 5.97 ± 3.47, according to a recent review by Bittar et al. [20] in Asia. China reported the shortest disease duration with a mean of 4.65 years, while India, Hong Kong, Taiwan, Korea, Japan, Thailand, and Singapore reported longer disease durations than this study. This shows that patients in Indonesia have shorter disease duration with high severity, and early identification of SpA may potentially lead to prompt and more effective intervention to delay disease progression and prevent future disability [21].

Our results showed that patients with early SpA had significantly higher serum DKK-1 levels than patients with established SpA. This result is in line with the study by Rubio Vargas et al. [3] which discovered that DKK-1 serum levels in people with axial SpA depend on the disease duration and decrease as the disease duration increases. Despite having high serum DKK-1 levels, patients with early SpA had significantly lower disease activity compared to patients with established SpA as indicated by lower values of both ASDAS-ESR and ASDAS-CRP. This supports the potential of DKK-1 as a strong diagnostic marker for early SpA. We assume that increasing serum DKK-1 levels can detect patients with early SpA even without visible progression in disease activity or bone radiography changes.

The disease activity was correlated with the DKK-1 level in the study by Descamps et al. [18]; this is in contrast with our results that reported only disease duration and BMD at the total hip were correlated with the serum DKK-1 level. There can be several reasons why the correlation of disease duration with some parameter can differ. One of the possible reasons is differences in study design, such as the sample size, inclusion criteria, and methods used to assess the disease and the parameter in question. Additionally, variations in patients' characteristics, such as age, sex, ethnicity, and comorbidities, can influence the correlation between disease duration and the parameter of interest. Variations in the statistical methods used to analyze the data, such as adjustments for confounding variables or the use of different correlation coefficients, can also contribute to differences in the reported correlations [22–24].

On the other hand, several studies reveal the similar result to our findings [9, 25, 26]. This could imply that the role of bone formation markers is complex and may differ in the regional area through different genetic and environmental influences. Wingless protein (Wnt) signalling and growth factors such as bone morphogenetic proteins (BMPs) are involved in the complex systems that underpin new bone formation in patients with axial SpA. The DKK-1 is a crucial inhibitor of Wnt signalling that appears to be boosted by tumour necrosis factor alpha (TNF-*α*) [3, 10]. The low DKK-1 expression caused inflammatory bone loss. The DKK-1 inhibits osteoblast differentiation, promotes sclerostin expression, and induces osteocyte death [9]. Besides becoming the potential marker for early SpA, DKK-1 inhibition at an early stage may thus be a powerful strategy for protecting bone from inflammatory damage.

Aside from new bone formation, the presence of syndesmophytes is one of the best predictors of radiographic spinal progression [27, 28]. Chronic spine inflammation causes not only new bone formation in the axial joints and vertebral spaces but also bone resorption, which leads to osteoporosis [29]. However, our findings showed that low BMD was not associated with disease duration or disease activity, as evidenced by no significant difference in the duration and ASDAS values of both ESR and CRP between patients with and without low BMD.

Lateral radiographs of the lumbar and cervical spine depicted by mSASSS showed no significant difference between early and established SpA patients. Kim et al. [12] found that the presence of low BMD and syndesmophytes at baseline were not associated with the formation of new syndesmophytes in patients with early axial SpA, which is consistent with the findings of this study. mSASSS in patients of this study with low BMD did not differ significantly from patients with normal BMD. The absence of statistical differences between patients with and without low BMD in terms of disease duration and disease activity might be attributed to the sample size of each group. However, our findings indicated that low BMD was not associated with either the disease duration or the disease activity in patients with spondyloarthritis, as there were no significant differences observed in the duration and ASDAS values of both ESR and CRP between patients with and without low BMD.

We suggest that low BMD is not a vigorous marker for early SpA detection because many factors, such as anti-inflammatory treatment, can influence the progression of patients with low BMD [12]. In spite of being unrelated to disease duration or activity, we discovered that low BMD was associated with patients' BMI; patients with low BMD have significantly lower BMI than patients with normal BMD. This finding was in line with Cai et al.'s study that found a positive relationship between BMD and BMI in axial SpA patients, but BMI has no effect on the disease activity in axial SpA [30].

The main limitations of this study include the small number of patients and the single-center scope. Additionally, the awareness of Indonesian society for SpA regular checkup made us lose contact with some patients, which made follow-up data collection challenging. As a cross-sectional study, we cannot draw definitive causal conclusions about the relationship between DKK-1 levels and disease duration. To provide a more comprehensive picture of DKK-1's potential as a marker in early SpA, future studies should include a larger number of participants, including those receiving different treatments. Moreover, a cohort study specifically in Indonesia could investigate the progression of DKK-1 levels in SpA patients and their overall role in the disease. Despite these limitations, the findings of this study provide groundwork for future research into the potential of DKK-1 as a marker and treatment target in early SpA.

## 5. Conclusion

Significantly higher serum DKK-1 levels were correlated with disease duration in patients with early spondyloarthritis, and the serum DKK-1 levels were also found to be correlated with disease duration. In addition, we found that low BMD at baseline was not related to disease activity measured with ASDAS-CRP and ASDAS-ESR, mSASSS, serum DKK-1 levels, or other patients' clinical parameters, except for BMI. However, BMD at the total hip was correlated with serum DKK-1 levels. Our study also revealed that patients with early SpA at Dr. Soetomo General Hospital had a relatively shorter disease duration and severe disease activity. Early diagnosis may lead to rapid and adequate treatment to delay disease progression and prevent future impairment. Although our study provides insights into the potential role of DKK-1 as a diagnostic marker or treatment target in early SpA, we recognize the need for larger scale studies to confirm and emphasize this potential.

## Figures and Tables

**Table 1:** Patients' characteristics.

Characteristics	Value (*n* (%)/mean ± SD/median (95% CI))
Sex (male: female)	15 (48.4) : 16 (51.6)
Age (years)	34.71 ± 8.59
BMI	22.48 ± 4.52
Underweight	7 (22.58)
Normal	12 (38.71)
Overweight	3 (9.68)
Obese	9 (29.03)
Disease duration (years)	5.97 ± 3.47
Early (≤5 years)	14 (45.16)
Established (>5 years)	17 (54.84)
Phenotype	
Axial	9 (29)
Axial and peripheral	22 (71)
ESR (mm/hour)	29 (20–61)
CRP (mg/L)	12.87 (2.4–14)
ASDAS-ESR	2.85 ± 1.12
ASDAS-CRP	2.43 ± 1.09
Serum DKK-1 level (pg/dL)	6265 ± 1863
Treatment	
DMARD	16 (51.61)
Non-DMARD	15 (48.39)

SD: standard deviation, CI: confidence interval, BMI: body mass index, ESR: erythrocyte sedimentation rate, CRP: C-reactive protein, ASDAS: ankylosing spondylitis disease activity score, mSASSS: modified stoke ankylosing spondylitis spinal score, DKK-1: dickkopf-related protein 1, and DMARD: disease-modifying antirheumatic drugs.

**Table 2:** Patients' characteristics differences based on disease duration.

Characteristics	Early SpA (≤5 years)	Established SpA (>5 years)	*p* value
Age (years)	34.14 ± 8.76	35.18 ± 8.70	0.745
BMI	22.50 ± 4.15	22.46 ± 4.93	0.983
ESR (mm/hour)	24 (11–44)	49 (27–82)	0.054
CRP (mg/L)	2.35 (1.7–8.8)	12.70 (4.6–26.6)	0.028^*∗*^
ASDAS-ESR	2.34 ± 0.96	3.28 ± 1.10	0.018^*∗*^
ASDAS-CRP	1.94 ± 0.98	2.83 ± 1.05	0.022^*∗*^
Serum DKK-1 level (pg/dL)	7365 ± 2067	5360 ± 1054	0.001^*∗*^

BMI: body mass index, ESR: erythrocyte sedimentation rate, CRP: C-reactive protein, ASDAS: ankylosing spondylitis disease activity score, and DKK-1: dickkopf-related protein 1; ^*∗*^indicates a significant value (*p* ≤ 0.05).

**Table 3:** Patients' characteristics differences in relation to low BMD.

Parameters	Low BMD at any site		*p* value
	Yes (*n* = 8)	No (*n* = 14)	
Age (years)	32.50 ± 6.590	38.79 ± 8.816	0.095
BMI	20.80 ± 3.76	25.21 ± 4.00	0.019^*∗*^
Duration (years)	7.87 (5–11)	6.071 (3–8)	0.082
ESR (mm/hour)	29.88 ± 24	42.57 ± 35.71	0.383
CRP (mg/L)	4.1 (1.7–26.8)	5.15 (2–14)	0.906
ASDAS-ESR	2.55 ± 1.04	2.77 ± 1.18	0.665
ASDAS-CRP	1.75 (1.1–3.9)	2.05 (1.3–3.4)	0.907
Serum DKK-1 level (pg/dL)	5640 ± 2064	6406 ± 1012	0.253
mSASSS	25.38 ± 3.159	25.79 ± 7.526	0.885

BMD: bone mineral density, BMI: body mass index, ESR: erythrocyte sedimentation rate, CRP: C-reactive protein, ASDAS: ankylosing spondylitis disease activity score, DKK-1: dickkopf-related protein 1, and mSASSS: modified stoke ankylosing spondylitis spinal score; ^*∗*^indicates a significant value (*p* ≤ 0.05).

**Table 4:** Correlation between DKK-1 levels and patients' clinical parameters.

Correlation parameters tested with serum DKK-1 level	*r*	*p*
Age	0.020	0.914
BMI	0.163	0.380
Duration	−0.370	0.040^*∗*^
ESR	0.027	0.887
CRP	−0.064	0.731
ASDAS-ESR	−0.0004	0.998
ASDAS-CRP	−0.044	0.814
mSASSS	−0.110	0.626
BMD at the lumbar spine	0.103	0.649
BMD at the femoral neck	0.290	0.190
BMD at the total hip	0.467	0.028^*∗*^

DKK-1: dickkopf-related protein 1, BMI: body mass index, ESR: erythrocyte sedimentation rate, CRP: C-reactive protein, ASDAS: ankylosing spondylitis disease activity score, BMD: bone mineral density, and mSASSS: modified stoke ankylosing spondylitis spinal score; ^*∗*^indicates a significant value (*p* ≤ 0.05).

## Data Availability

All data used to support the findings of this study are available from the corresponding author upon request.
